# Remaining Useful Life Prediction with Similarity Fusion of Multi-Parameter and Multi-Sample Based on the Vibration Signals of Diesel Generator Gearbox

**DOI:** 10.3390/e21090861

**Published:** 2019-09-03

**Authors:** Shenghan Zhou, Xingxing Xu, Yiyong Xiao, Wenbing Chang, Silin Qian, Xing Pan

**Affiliations:** 1 School of Reliability and Systems Engineering, Beihang University, Beijing 100191, China; 2 Nanjing Research Institute of Electronics Technology, Nanjing 210039, China

**Keywords:** remaining useful life (*RUL*), similarity fusion, dynamic time warping, damage indicators extraction, approximate entropy variance, vibration monitoring

## Abstract

The prediction of electrical machines’ Remaining Useful Life (*RUL*) can facilitate making electrical machine maintenance policies, which is important for improving their security and extending their life span. This paper proposes an *RUL* prediction model with similarity fusion of multi-parameter and multi-sample. Firstly, based on the time domain and frequency domain extraction of vibration signals, the performance damage indicator system of a gearbox is established to select the optimal damage indicators for *RUL* prediction. Low-pass filtering based on approximate entropy variance (Aev) is introduced in this process because of its stability. Secondly, this paper constructs Dynamic Time Warping Distance (*DTWD*) as a similarity measurement function, which belongs to the nonlinear dynamic programming algorithm. It performed better than the traditional Euclidean distance. Thirdly, based on *DTWD*, similarity fusion of multi-parameter and multi-sample methods is proposed here to achieve *RUL* prediction. Next, the performance evaluation indicator *Q* is adopted to evaluate the *RUL* prediction accuracy of different methods. Finally, the proposed method is verified by experiments, and the Multivariable Support Vector Machine (*MSVM*) and Principal Component Analysis (*PCA*) are introduced for comparative studies. The results show that the Mean Absolute Percentage Error (MAPE) of the similarity fusion of multi-parameter and multi-sample methods proposed here is below 14%, which is lower than *MSVM*’s and *PCA*’s. Additionally, the *RUL* prediction based on the *DTWD* function in multi-sample similarity fusion exhibits the best accuracy.

## 1. Introduction

As a nonlinear dynamical system, a diesel generator’s safe and smooth running is essential to the reliability of systems. The gearbox is a core part of a diesel generator, directly determining its performance. Remaining Useful Life (*RUL*) prediction can detect faults early and estimate the downtime of diesel generator components, further helping operators to arrange a reasonable maintenance schedule and save operating costs.

Vibration signal analysis is one of the most widely used methods of condition monitoring. Vibration monitoring generally involves arranging sensors at important locations, using the data acquisition card to obtain signals, and finally using the computer to calculate and analyze the data. This article aims at analyzing the degradation trend of machines and predicts their *RUL* with the vibration signal collected from the sensors online or offline. In this way, the *RUL* of a diesel generator is achieved during condition monitoring.

**Figure 1:**
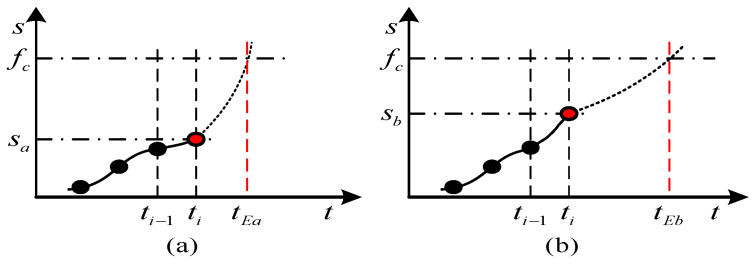
Performance of the system.

One of condition-based maintenance (*CBM*)’s main missions is to predict a machine’s *RUL* [1]. *RUL* prediction counts more than fault diagnosis in the makings of maintenance decisions [2]. According to the data and continuous degradation trend recorded by the condition detection system, *RUL* is predicted. It will forecast a potential degradation when current faults have been cleared, providing direct references for *CBM*. As Figure 1 shows, the functional degradation of *a* and *b* stands at an even level at *t_i−_*_1_. *S_a_*, *S_b_* represent the degree of performance degradation for machine *a* and *b*, and *f_c_* means that the machine is incapable of working. Additionally, at *t_i_*, *a*’s health level is higher than *b*’s, indicating that *a* is healthier. After *t_i_*, *a*’s function degrades faster than *b*’s and *a*’s *RUL* is shorter. Any planned maintenance must be performed on *a* in advance [3].

*RUL* is defined as the time span from the present moment to the end of the useful life [4], expressed as $l_{k}=t_{Eol}-t_{k}$, where *t_Eol_* is life termination, *t_k_* is the present moment, and $l_{k}$ is the remaining life at *t_k_*.

The primary mission of *RUL* prediction is to monitor the useful time left before the system loses its working capability according to condition detection information. Based on time series analysis, the accuracy of prediction is the primary factor considered in the choice of prediction method. The existing methods are based on physical models, statistical data, and artificial intelligence [5], as described in the following:

(1) *RUL* prediction methods based on physical models reflect the life-cycle degradation process of the system by establishing a mathematical model based on the failure mechanism [5]. As a typical physical model, the Paris-Erdogan model (PE) is widely used for *RUL* prediction. Frank et al. [6] used PE to predict the *RUL* of two types of pipelines, 80 and 100. Hu et al. [7] used Norton’s law to describe the creep of a turbine and combined the Kalman filtering (*KF*) and particle filter (*PF*) to predict *RUL*; however, the methods based on physical models need the deep understanding and sufficiently accurate judgment of failure mechanism to ensure the accuracy of RUL estimation.

(2) *RUL* prediction methods based on statistical data fit the observational data into a random coefficient model and a stochastic process model. This method is widely applied as many on-the-shelf statistical models can be applied to fit the data, that is for instance random coefficient models, autoregressive models, Gamma process models, inverse gaussian processes, Markov models, and proportional hazards models. However, Autoregressive models rely heavily on high-quality historical data and are not conducive to RUL prediction under complex operating conditions, Wiener models and Gamma process models is limited by the assumption of Markov, which assume that the future state is only related to the current state but not to the past state, so it is not applicable to some practical situations.

(3) *RUL* prediction methods based on artificial intelligence concentrate on learning the degradation pattern of the system from observations. Common *AI* techniques include the artificial neural network (*ANN*), neural fuzzy (*NF*), support vector machine/relevance vector machine (*SVM/RVM*), K-nearest neighbor (*KNN*) and Gaussian process regression (*GPR*). Hussain et al. [8] extracted the index of health from the vibration signal, and established the *RUL* prediction model by the adaptive neural fuzzy inference system and nonlinear autoregression. The *NF* excels in *RUL* prediction because it takes advantage of expert knowledge and intelligent *ANN*, but needs high-quality data sources. There are many different kinds of *SVM* that are used for machines’ *RUL* prediction, like one-class *SVM* and multi-class *SVM* [9], and Squares-*SVM* [10]. However, *SVM* only provides point estimate and does not provide a probability distribution over of points. In order to make up for this shortcoming, *RVM* was proposed, which has the same functional form as *SVM*, but provides a full probability distribution over all possible outcomes [11]. However, those methods focus more on data training rather than analysing the mechanism of mechanical failure. The structure and parameters of *ANN* need to be set artificially, which leads to low generalization ability; Kernel function selection for SVM/RVM with different objects is a huge challenge. Calculation process of GPR is complex and takes a long time.

It can be seen from the above that the three *RUL* prediction ideas have their own limitations. The methods of *RUL* prediction are variable, among which, similarity measure of the data-driven prediction is advantageous at avoiding constructing complex functional degradation models. Therefore, this paper will study *RUL* prediction based on statistical data from the perspective of similarity measure. Research on similarity-based *RUL* prediction was first proposed in 2012 and has been proved to be a very effective RUL prediction approach [12,13,14,15,16]. However, the methods have not been so widespread until now. The basic idea is that products with similar degradation processes have a similar service life [3]. The *RUL* of the test sample is determined by observing the similarity between the performance degradation trajectory of the test sample and the reference samples of the known life-cycle degradation process.

There is little literature about RUL prediction based on similarity but they verified the validity of “similarity” idea. You et al. [12] conducted an experiment to predict the *RUL* of a welding spot under vibrations. He thought if the asset under study is more similar to reference sample “A”, then “A” should play a more important role in RUL estimation of the asset under study. Eker [13] testified the function of similarity-based prediction through data collected from Virkler’s fatigue crack propagation, a degradation data set of drilling, and a turnout system of slide chair degradation. Zhang [14] put forward a method to predict the *RUL* of a mechanical system based on the similarity of a phase space trajectory and found that the results approximated the actual *RUL* very closely. Xiong [15] built a one-dimensional damage indicator on an aero engine’s multiple parameters by means of liner regression. He obtained the *RUL* after matching test engine data to the model base. In the same way, Moghaddass [16] adopted principal components analysis to integrate a turbine engine’s multiple parameters and drew the first principal component to describe the system degradation process.

It can be concluded from the literature review that similarity-based *RUL* prediction methods so far are almost always built on a single parameter. The latest research is only employed to integrate multiple parameters into a one-dimensional parameter firstly, and then compare the similarity of performance degradation curves with statistical methods or *AI* methods. There is no research about co-impact both multiple samples and multiple parameters of those samples on *RUL* prediction. However, performance degradation or malfunction may result from a multitude of reasons. Thus, multiple parameters of different perspectives may provide a more comprehensive reflection of the running process [17]. Especially for a complex system, what a single parameter can present is far less than multiple parameters in describing the degradation of various forms.

Therefore, this paper proposes an *RUL* prediction method based on the similarity fusion of multiple damage indicators and samples. In contrast to the more traditional methods, the method of multi-parameter and multi-sample similarity fusion estimates *RUL* by referring to multiple parameters and samples.

The process can be divided into five parts. At first, in Section 2.1 and Section 2.2, the various time and frequency domain features extracted from a vibration signal that will be applied as damage indicators are introduced together with the entropy variance method for fuzzy filtering applied for low pass filtering of the time-domain features. Further, the method used for parameter evaluation in order to select the most significant performance damage indicators to be applied for RUL prediction is discussed. Second, in Section 2.3, we introduce principles of *RUL* prediction based on similarity and defines four core elements in the *RUL* prediction based on similarity: Time window *D*, similarity measurement function *S*(.), weight function *w*(.), and performance evaluation indicator *Q*. Third, in Section 2.4, we introduce the Dynamic Time Warping Distance (*DTWD*) as the similarity measure function *S*(.) to discuss the similarity of data degradation trajectory patterns for the first time. Fourth, in Section 2.5, Section 2.6 and Section 2.7, according to combinations of different performance damage indicators, the *RUL* prediction model based on the similarity fusion of multi-parameter and multi-sample methods is established. Finally, in Section 3, this paper studies a type of heavy high-speed diesel generator produced by the China Shipbuilding Industry Corporation (*CSIC*), and validates the *RUL* prediction method proposed here with experimental results. In the meanwhile, Proposed method here are compared with the mature methods of Multivariable Support Vector Machine (*MSVM*) and Principal Component Analysis (*PCA*) for comparison analysis in Section 3.3 and Section 3.4.

## 2. Methodology

At first step, we will select the most significant performance damage indicators which will be the input of RUL estimation from various time and frequency domain features. Then, we will define four core elements in the RUL prediction based on similarity: Time window D, similarity measurement function S(.), weight function w(.), and performance evaluation indicator Q. Next, as the most important core, similarity measurement function S(.) will be established with DTWD and we write the details about DTWD in Section 2.3. At last, the RUL prediction model based on the similarity fusion of multi-parameter and multi-sample methods will be established.

### 2.1. The Damage Indicators

The various time domain and frequency domain features extracted from the vibration signal will be used as damage indicators in the following RUL prediction. Further, we discuss the method we apply to to define for each individual gearbox under study a subset of most significant damage indicators system, to be applied for RUL prediction for this particular gearbox. The time domain features of the vibration signal effectively reflect the performance degradation of the gearbox [18]. As shown in Table A1 of Appendix A, we have chosen to use 10-time domain features as damage indicators [19]. Further, the Fourier transform is applied to convert the vibration signal into its frequency spectrum representation [20]. We have chosen to use 15 frequency domain features [21], as damage indicator, see Table A2 in Appendix A.

Since the time-series of the various damage indicators are noisy and in order to correctly compare them with the reference samples we need to smooth the series, i.e., low pass filtering. Fuzzy filtering is a low pass filtering method based on fuzzy set theory, which can adjust the filter structure adaptively based on the features of the signal [22]. A large number of studies have shown that this method is easy to implement and has a good filtering effect, which is very suitable for engineering applications.

For time domain features, this paper proposed the low pass filtering based on approximate entropy variance. The time-series of the various damage indicators are rather noisy, we apply low pass filtering techniques to smooth them [23]. For the time domain damage indicators we have applied low-pass filtering with approximate entropy variance (Aev), because approximate entropy [24] is suitable for describing dynamic noise with a small amount of data and has a strong Robustness to observation noise, and the dynamics system is easy to reconstruct. Approximate entropy variance is a statistic measuring the complexity of time series and it can accurately measure the complexity of signals. Especially in the case of small data quantity and noise interference, it also demonstrates statistical stability. The variance could describe the stability in time series. Approximate entropy (*Ae*) is defined as: For time series {n(i)}(i=1,2,…(N), *x*(*i*) denotes *m* consecutive values of *u* starting at point *i*:

(1) $$Ae(m,r)=\underset{n\to \infty }{\lim}\left[\varphi^{m}(r)-\varphi^{m+1}(r)\right]$$

where:

(2) $$\varphi^{m}(r)={\left(N-m+1\right)}^{-1}\sum_{i=1}^{N-m+1}\left[\sum_{j=1}^{N-m+1}H\left\{r-d_{m}\left[x_{m}(i),x_{m}(j)\right]\right\}/(N-m+1)\right]$$

(3) $$\begin{array}{l} d_{m}\left[x_{m}(i),x_{m}(j)\right]=\max\left|u(i+k)-u(j+k)\right|\\ x_{m}(i)=\left[u(i),u(i+1),\ldots ,\;(u(i+m-1)\right] \end{array}$$

*H*() is the Heaviside function, After *Ae* is calculated, *Aev* is defined as:

(4) $$\mathrm{Aev}=\sum_{i=1}^{N}(\mathrm{Ae}-\bar{\mathrm{Ae}})/N$$

Then low-pass filtering decomposes the damge indicator signal into the parts trend and noise:

(5) $$X\left(t_{k}\right){=X}_{T}\left(t_{k}\right){+X}_{R}\left(t_{k}\right)$$

With *X*(*t_k_*) is the value of the performance damage indicator at time *t_k_*, *X_T_*(*t_k_*) is the trend term, *X_R_*(*t_k_*) the noise term, and *t_k_* = 1, 2,…, *N*, with *N* the number of discrete observations made within the measurement time interval.The weighting filter and fuzzy filtering membership function are defined as $u^{\prime }(x_{n-k})=f(Ae,n-k)$ according to [24], the range of u’(x_n−k_) is a [0, 1], and *f* is set to normal distribution function. So XT(t_k_) will be ramained while XR(t_k_) removed.

To smooth the frequency domain damage indicator over time, a simple moving average filtering is applied. The moving average filtering can reduce random noise while reflect unit step function response of signal [25]. First, the damage indicators are decomposed into two parts just as before in Equation (5), then calculate the average value as the predicted value of the next sub-interval and move forward in turn. $\hat{X}\left(t_{j}\right)$ is the first part of damage indicator with moving average filtering which is defined as the weighted average value of the adjacent *N* data points.

(6) $$\hat{X}\left(t_{j}\right)=\frac{1}{n}\sum_{i=j-n}^{j-1}X\left(t_{i}\right)\;j=n+1,n+2,L,N+1$$

**Figure 2:**
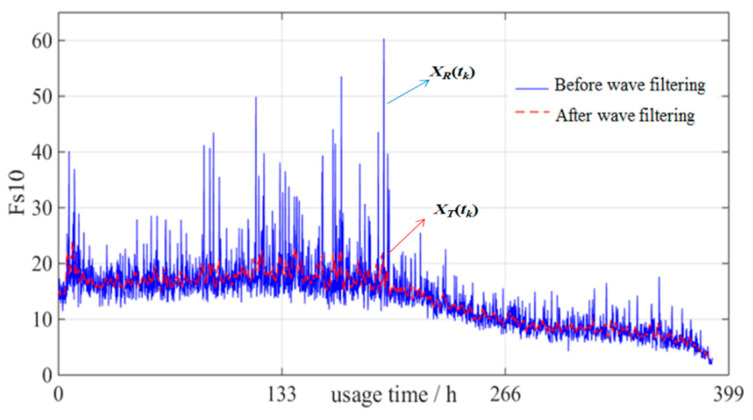
Curves comparison of *F_S_*_10_ before and after wave filtering.

In Figure 2, as an example of the full signal together with its trend is shown for one of the time/frequency domain indicators. The ideal output can be obtained by wave filtering.

### 2.2. Defining a Subset of Most Significant Damage Indicators

To define an—asset dependent—subset of most significant damage indicators to be applied for RUL prediction of the asset, so called significance indicators have been defined [26]. By aid of these significance indicators each of the twenty-five damage indicators is evaluated and a score from 0 to 1 is given to each damage indicator as a measure of how significant the parameter is for the RUL prediction for the asset under study. We have defined three significance indicators, Correlation, Monotonicity and Robustness to act as RUL significance indicators, and which will be defined and explained in the following.

The correlation *r* measures the correlation of a damage indicator with time (that is over the whole time span the vibration measurements have been performed), i.e., it states the normalized slope of the trend of the damage indicator over time, i.e., r=(σX/σt)b, with X is the damage indicator, *σX* the standard deviation of *X*, *σt* the standard deviation of the the variable t time, and *b* the slope of the regression line ‘found by linear regression when viewing *X* as a function of *t*.

The Monotonicity indicator reflects the unidirectional trend of time domain features and frequency domain features. The larger the value of Monotonicity, the greater the slope of the parameters, and the more intuitive and obvious the trend of performance degradation. If a parameter rises and falls recurrently in the degradation process, it may be just a cyclic change as the machine vibrates. That does not change in a certain direction as performance degradation occurs.

The Robustness indicator reflects the tolerability of damage indicators for outliers. Robustness measures whether the degradation parameter is capable of resisting random interference [27]. If a parameter is sensitive to external disturbance, it does not contain valuable information even if it fluctuates wildly.

The equations applied to compute each of the indicator indicators are stated in Appendix B.

This study proposes a combination function W with three indicators above as a “ruler” to select several optimal parameters for following RUL prediction.

(7) $$\underset{X\in \Omega }{\max}{W=\omega }_{1}{\mathrm{Corr}(x)+\omega }_{2}{\mathrm{Mon}(X)+\omega }_{3}\mathrm{Rob}(X)\;\mathrm{with}:\;\omega_{i}>0\cap \sum_{i}\omega_{i}=1\;i=1,2,3$$

In this equation, *W* is the combination function, distributed in the range of [0, 1]; *Ω* represents a set of candidate damage indicators; and *ω_i_* represents the weight of each indicator. The parameter with a larger value of *W* should be selected for effective *RUL* prediction. *ω_i_* is determined by two sources: Subjectively, due to the fact that damage indicator is used to describe performance degradation trajectory as time goes, *Mon* should take up the largest weight. This is in compliance with similarity-based prediction method. So *ω_i_* will be subjectively assigned a value denoted as prior weighing *a_i_*. While objectively, the optimal combination of the chosen damage indicators in essence is about constrained optimization. We adopt the solving model with *AMPL*, input the permutation and combination of three indicators’ weights (adjustment of weighting is from 0.2~0.8), and determine the posterior weighting *b_i_*. according to the results. At last, considering both prior weighing *a_i_* and posterior weighting *b_i_*, *ω_i_* will be determined, and some more significant damage indicators can be chosen for subsequent *RUL* estimation.

(8) $$\omega_{i}=\alpha a_{i}+(1-\alpha )b_{i},\;(0\leq \alpha \leq 1)$$

### 2.3. Similarity-Based RUL Prediction

**Figure 3:**
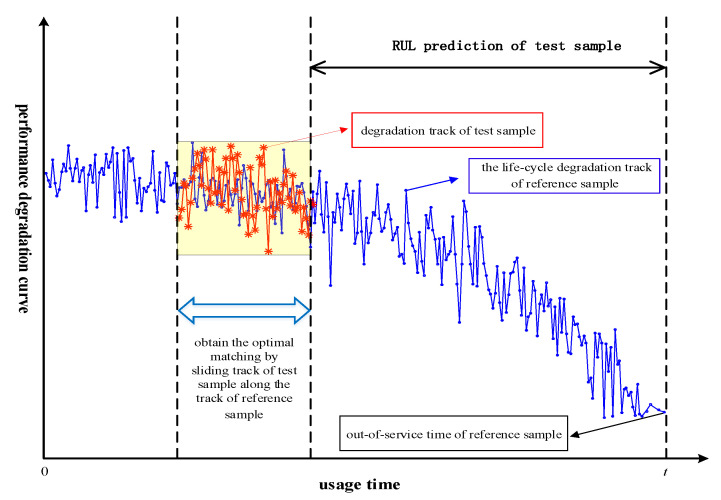
Principle of similarity-based Remaining Useful Life (*RUL)* prediction.

As Figure 3 shows, the concept of the similarity-based RUL prediction method is that assets that show similar behavior of their damage indicators have similar RUL values [28]. By comparing the damage indicator time series of an asset with corresponding historical reference time-series, the RUL of the asset can be predicted. It is assumed that the assets from which reference indicator curves are available are the same or of closely related type of product or system—and have performed under more or less similar operating environments and conditions—as the asset under study.

The blue curve represents the time-series of one of the damage indicators over time for a reference gearbox, while the red curve is the time-series of same indicator for a gearbox in use on which we wish to make an RUL prediction. Now the similarity concept states that we should find the most similar certain part of blue curve to red curve, which named ‘optimal match’. When an optimal match has been established then as estimate for the RUL of gearbox of interest the length of the time interval of the blue curve which is on the right of the red is applied. Here we always assume that the final available measurement point of any of the reference curves corresponds with the end of the remaining useful life of the reference gearbox.

To apply the similarity prediction method, its four core elements need to be defined. These are the time window *D*, the similarity measure function *S*(.), a weight function *w*(.), and the performance evaluation indicator *Q*. The time window *D* refers to the time interval of similarity between the test sample and reference samples, shown as the data block length marked as yellow in Figure 3. The similarity measurement function *S*(.) quantifies the similarity of the degradation trajectory of the test sample and reference samples. This paper will establish the *DTWD*-based nonlinear dynamic programming algorithm as *S*(.) which will be explained in Section 2.4. The weight function *w*(.) concerns the similarity between the test sample and reference samples, and it gives different weights to different reference samples and different parameters in line with their contributions. The performance evaluation indicator *Q* is used to describe differences between the *RUL* estimated value and its actual value, which helps to find the optimal method through comparing different *RUL* prediction methods. We borrowed 5 indicators as the performance evaluation indicator *Q*, which are shown in Appendix B (2).

Similarity-based RUL prediction follows four steps:

(1) Define the time window *D* to be used for each of the damage indicators related to an asset. The right side of the data block is the state of asset under study. The red curve is the time window D of the test sample and the blue curve is the life-cycle degradation state of reference sample. The right boundary line of D is observation point at present for test sample.
(2) Define a similarity measure function S(.) through which the similarity or closeness between two time-series is defined. *DTWD* algorithm is established as the similarity measure function S(.) in order to find the most similar part in one certain reference asset with the time window *D*, so one similarrity distance could be obtained. Suppose *H* most significant damage indicators are selected and L reference assets are compared with the asset under study, which means each reference asset contains *H* damage indicators. Then H*L similarity distances between each damage indicator in the asset under study and each damage indicator in those reference assets could be obtained by DTWD algorithm.
(3) Based on the thought “the more similar the two-time series is, the larger the weight value is”, we will make weighted summation among those H*L similarity distances. That is normalizing H*L similarity distances and then assigning different weights according to the thought such as closer distances will be given greater weights. The details of weight function *w*(.) based on multi-parameter and multi-sample refers to Equations (12) and (14) and Equations (16) and (18), respectively.
(4) For those RUL values referring to different parameters or different samples, weighted average method is used to obtain the test sample’s RUL estimation based on the corresponding weights calculated in step (3).

### 2.4. Similarity Measurement Function S(.): Dynamic Time Warping Distance (DTWD)

**Figure 4:**
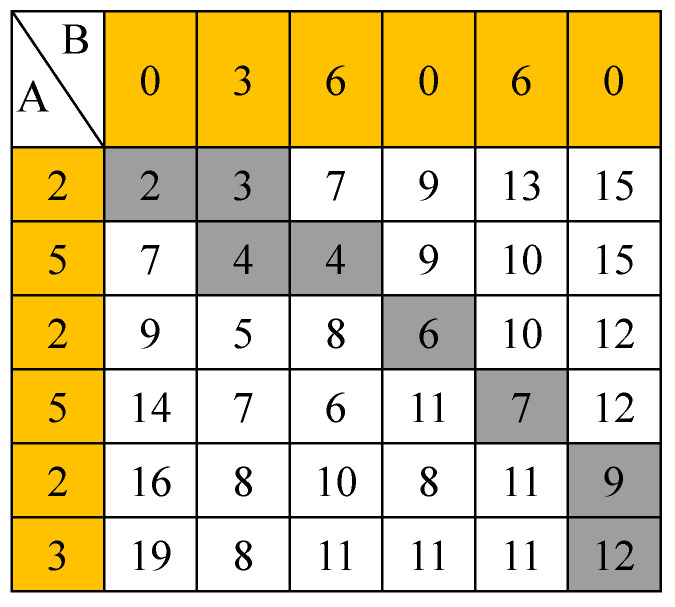
The dynamic time warping path.

The *DTWD* is a dynamic nonlinear programming idea, and an algorithm that matches time dimension warping with distance optimization planning [29]. DTWD has been widely used in text data matching, voice information processing and other fields in recent years. Compared with the traditional Euclidean distance, it shows better recognition accuracy and robustness in the application of time series. DTWD can compress and bend time series, make the overall distance of two sequences smaller. The DTWD of two time series is defined as the minimum distance between the two series calculated by time dimension bending. when calculating the distance between series A and B, traditional Euclidean Distance takes the distance between two time series A and B at same time point, while DWTD takes the distance between two time series A and B that needn’t at same time point in order to obtain the shortest distance. For example, supposing that time series A = {2,5,2,5,2,3}, B = {0,3,6,0,6,0}, so the traditional Euclidean Distance is calculated as 2 + 2 + 4 + 5 + 4 + 3 = 20, and DTWD is calculated as Figure 4. The gray elements from the upper left corner to the lowest right corner are dynamic time warping path. The lowest right corner element ”12” is the cumulative distance D_twd_(A,B) = 12.

Therefore, *DTWD* is calculated as follows: Setting time series *A =* (*a*_1_*,a*_2_*,…,a_l_)* and *B =* (*b*_1_*,b*_2_*,…,b_j_…,b_k_*), *l* and *k* represent the sequence length of *A* and *B*, respectively. The *DTWD* algorithm needs to first align two time series and establish a *l × k* matrix *D* which contains the value d(*a*_i_,*b*_j_) on its ij-th entry. *d* (*a_i_*,*b_j_*) represents the distance between points *a_i_* and *b_j_* in two time series.

In matrix *D*, *P* (P = *q*_1_,*q*_2_,…,*q*_n_,…,*q*_N_) denotes the dynamic time warping path of time series *A* and *B*, *q_i_* represents the distance of time series A and B at time point i. Path *P* needs to meet the following four restraint conditions:

(1) Boundedness: max(*l*,*k*) < N < l + k_−1_;
(2) Boundary conditions: q_1_ = D(1,1) and q_N_ = D(*l,k*), that is, the start and end points of the dynamic warping path can only be on the diagonal of the matrix;
(3) Continuity: For *q*_n_ = (*a,b*) and *q*_n−1_ = (*a’,b’*), the conditions $a-a^{\prime }\leq 1$ and $b-b^{\prime }\leq 1$ must be met;
(4) Monotonicity: For *q*_n_ = (*a,b*) and *q*_n−1_ = (*a’,b’*), *a* − *a*’ = 0 and *b* − *b’* = 0 can’t happen. that is, all line segments representing the dynamic bending paths cannot intersect each other.

For small-scale data, an exhaustive search method can be used to find an optimal dynamic time warping path. For large-scale data, based on the Dynamic Programming Model, the optimal dynamic time warping path can be obtained by a recursive search algorithm with the local optimal solution from point (1,1) to point (*i*,*j*). Using *DTWD* to represent *DTWD* between time series *A* and time series *B*, the computation process is

(9) $$\left\{ \begin{array}{l} D_{\mathrm{twd}}\left(A,B\right)=d\left(a_{1}{,b}_{1}\right)+\min\left\{ \begin{array}{l} D_{\mathrm{twd}}\left(A,\mathrm{rest}\left(B\right)\right)\\ D_{\mathrm{twd}}\left(\mathrm{rest}\left(A\right),B\right)\\ D_{\mathrm{twd}}\left(\mathrm{rest}\left(A\right),\mathrm{rest}\left(B\right)\right) \end{array}\right.\\ d\left(a,b\right)={\left\|a-b\right\|}_{p} \end{array}\right.$$

In the equation, *p* denotes the norm, rest(A) = {*a*_2_*, a*_3_…*a_l_*}, rest(B) = {*b*_2_*, b*_3_…*b_k_*}. As Equation (9) showed, *d(a_i_*,*b_j_)* represents the first point’s distance between two time series, then search for each shortest bending path at each rest point(i.e., rest(A) and rest(B)) between two series. The pseudocode of *DTWD* algorithm is shown in Appendix B (3).

### 2.5. RUl Estimation by Multi-Parameter Fusion

Multi-parameter similarity fusion focuses on the impact of different parameters on the RUL estimation of the asset under study. As the four steps showed in Section 2.3, Suppose *H* most significant damage indicators are selected and *L* reference assets are compared with the asset under study, which means each asset contains *H* damage indicators. Then H*L similarity distances between each damage indicator in the asset under study and each damage indicator in those reference assets could be obtained by *DTWD* algorithm. First, according to the weight idea in step (3) of Section 2.3, different weights are arranged to those H*L similarity distances. Second, for each certain damage indicator H*i*, we make weighted summation among those *Hi* from *L* reference assets respectively, which is called “first fusion” and need to be traversal *H* times because there are total of *H* damage indicators. After first fusion there will be *H* similarity distances formed. Third, for those formed *H* similarity distances, we make weighted summation among them again based on the weight idea in step (3). This is called “second fusion”. There will be one similarity distances formed called “RUL value”. At last, by finding the corresponding time point of “RUL value”, we can estimate the RUL.

**Figure 5:**
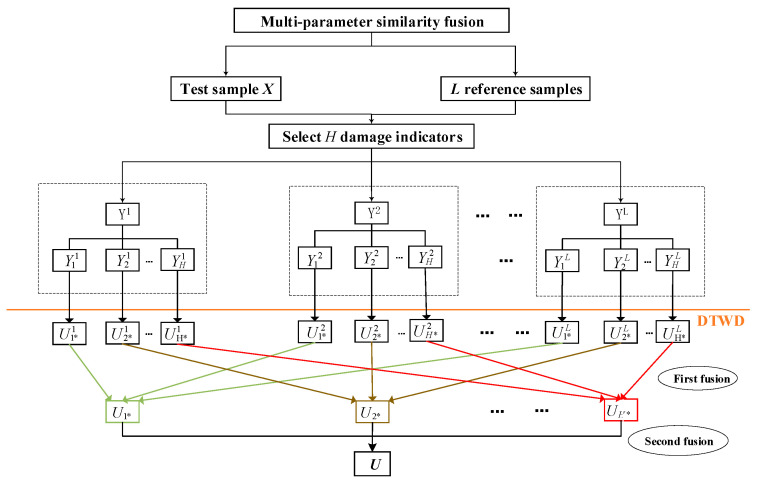
Similarity fusion of multi-parameter.

The following is the calculation process of mathematical theory:

For a diesel generator gearbox, with a asset under study(called “test sample’) of *X*, suppose *H* performance damage indicators can be obtained with the method in Section 2.2. With the *l*-th reference sample *Y^l^*, *l* (l=1,2,⋯,L) is the label of reference sample and *L* is the number of reference samples. The idea of multi-parameter similarity fusion is shown in Figure 5.

In Figure 5, by $y_{h}^{l}$ we denote the time-series of the *h*-th damage indicator of the *l*-th reference gearbox, with *l* = 1,…, *L*, and *L* the total number of reference gearboxes. Further, $U_{h*}^{l}$ represents the RUL estimation described by the *h*-th damage indicator of the *l*-th reference sample, and $U_{h*}$ represents the *RUL* value estimated by the *h*-th damage indicator after first fusion.

(1) Calculating similarity distance between each damage indicator in the asset under study and each damage indicator in those reference assets which is denoted by $U_{h*}^{l}$, so we need to run this step H*L times and obtain total of H*L similarity distances. let $S_{h*}^{l}$ denotes the optimal similarity distance by DTWD between the *h*-th damage indicator of the *l*-th reference sample and the *h*-th damage indicator of the test sample. The calculation of $U_{h*}^{l}$ is as follows:

(10) $$S_{h*}^{l}=\underset{\Delta }{\min}\;D_{twd}\left(x_{h*},y_{h*}^{l}\left(N-D-\Delta \right)\right)$$

(11) $$U_{h*}^{l}=\arg\;\underset{\Delta }{\min}\;D_{twd}\left(x_{h*},y_{h*}^{l}\left(N-D-\Delta \right)\right)$$

As Figure 3 shows, we need to match the red block such that is most similar (w.r.t. a certain measure) to a part of the blue curve, that is to find an ‘optimal match’. Only when the *D_twd_* attains a minimum, we can conclude that the right boundary line of D which corresponds to a time point of reference sample reflects the RUL of test sample. With the minimum of distance $S_{h*}^{l}$ is determined, the $U_{h*}^{l}$ is determined.

**Figure 6:**
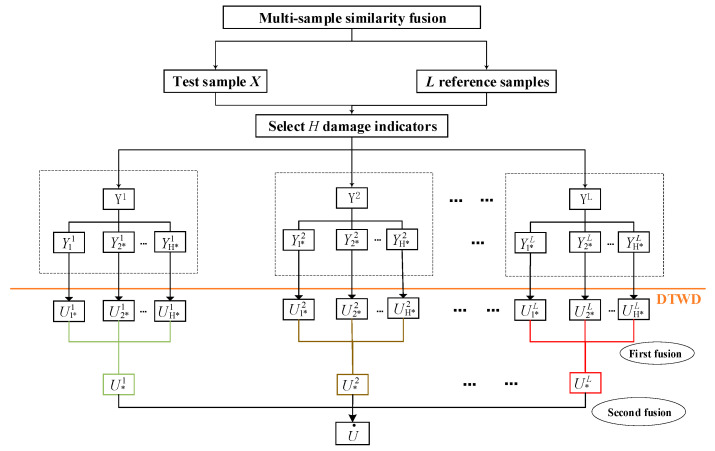
Similarity fusion of multi-sample.

(2) First fusion:$w_{h*}^{l}$ represents the weight of $U_{h*}^{l}$, so Equation (12) is established as weight function w(.) for the first fusion according to the idea” The smaller the distance between the two time series is, the larger the weight value of the parameter is.”, then $U_{h*}^{}$ could be obtained as showed in Figure 6. $S_{h*}^{l}$ and $U_{h*}^{l}$ have been calculated in Equations (10) and (11).

(12) $$w_{h*}^{l}=\frac{\sum_{l=1}^{L}S_{h*}^{l}}{\sum_{l=1}^{L}(\sum_{l=1}^{L}S_{h*}^{l}/S_{h*}^{l})\cdot S_{h*}^{l}}$$

(13) $$U_{h*}^{}=\sum_{l=1}^{L}w_{h*}^{l}\cdot U_{h*}^{l}$$

(3) Second fusion: After obtaining a total of *H* $U_{h*}$, $w_{h}$ represents the weight of $U_{h*}$, so Equation (14) is established as weight function w(.) for the second fusion.

(14) $$w_{h}=\frac{\sum_{h=1}^{H}\sum_{l=1}^{L}S_{h*}^{l}}{\sum_{l=1}^{L}(\frac{\sum_{h=1}^{H}\sum_{l=1}^{L}S_{h*}^{l}}{\sum_{l=1}^{L}S_{h*}^{l}})\cdot \sum_{l=1}^{L}S_{h*}^{l}}$$

(15) $$U=\sum_{h=1}^{H}w_{h}\cdot U_{h*}$$

### 2.6. RUl Estimation by Multi-Sample Fusion

Compared with multi-parameter similarity fusion, multi-sample similarity fusion focuses more on the similarity between reference assets and the asset under study, rather than the similarity among different parameters. Same as Section 2.5, suppose *H* most significant damage indicators are selected and *L* reference assets are compared with the asset under study, which means each asset contains *H* damage indicators. Then H*L similarity distances between each damage indicator in the asset under study and each damage indicator in those reference assets could be obtained by *DTWD* algorithm. First, according to the weight idea in step (3), different weights are arranged to those H*L similarity distances. Second, for each certain reference sample *L_i_*, we make weighted summation among those *Hi*, which is called “first fusion” and need to be traversal *L* times because there are total of *L* reference samples. After first fusion there will be *L* similarity distances formed. Third, for those formed *L* similarity distances, we make weighted summation among them again based on the weight idea in step (3). This is called “second fusion”. There will be one similarity distances formed called “RUL value”. At last, by finding the corresponding time point of “RUL value”, we can estimate the RUL.

**Figure 7:**
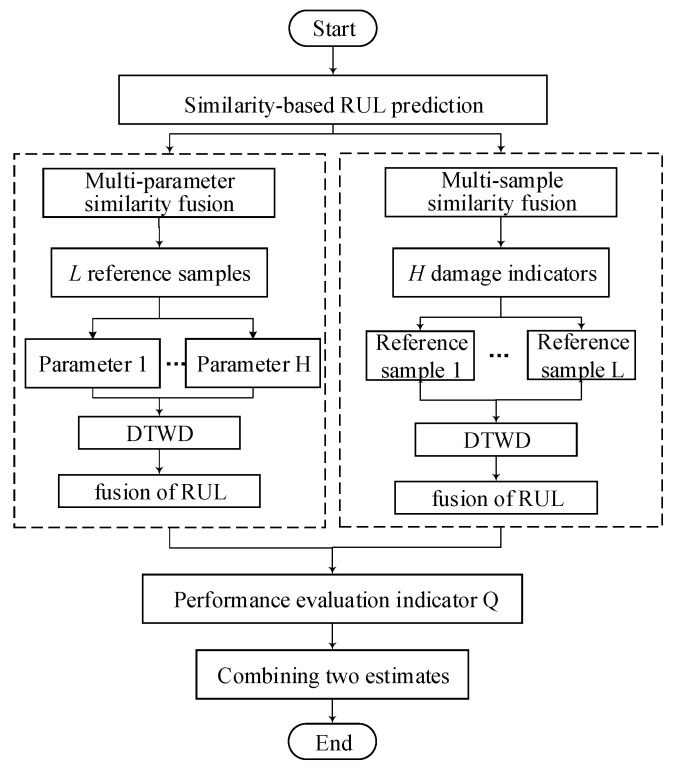
Combination of the two methods.

The following is the calculation process of mathematical theory:

(1) Repeating the steps (1) in Section 2.5 based on multi-parameter similarity fusion;
(2) First fusion: $\overset{•}{w_{h*}^{l}}$ represents the weight of $U_{h*}^{l}$, so Equation (16) is established as weight function w(.) for the first fusion, then $U_{h*}^{}$ could be obtained as showed in Figure 7. Unlike multi-parameter fusion, each reference sample is treated as a “unit”, *H* damage indicators of a certain reference sample will have a fusion firstly in those units.
  (16) $$\overset{•}{w_{h*}^{l}}=\frac{\sum_{h=1}^{H}S_{h*}^{l}}{\sum_{h=1}^{H}(\sum_{h=1}^{H}S_{h*}^{l}/S_{h*}^{l})\cdot S_{h*}^{l}}$$
  (17) $$U_{*}^{l}=\sum_{h=1}^{H}{\dot{w}}_{h*}^{l}\cdot U_{h*}^{l}$$
(3) Second fusion: For the obtained $U_{*}^{l}$, We used Equation (18) as weight function w(.) and make weighted summation to integrate L reference sample to the final RUL value $\overset{•}{U}$:
  (18) $$w^{l}=\frac{\sum_{h=1}^{H}\sum_{l=1}^{L}S_{h*}^{l}}{\sum_{l=1}^{L}(\frac{\sum_{h=1}^{H}\sum_{l=1}^{L}S_{h*}^{l}}{\sum_{h=1}^{H}S_{h*}^{l}})\cdot \sum_{h=1}^{H}S_{h*}^{l}}$$
  (19) $$\overset{•}{U}=\sum_{l=1}^{L}w^{l}\cdot U^{l}$$

### 2.7. Combining the Two Estimates into One

After obtaining the two results of RUL estimation with two methods, it’s feasible to make “third fusion” to combining the two estimates into one. This paper provides another idea about combining yet. As Figure 7 showed, the performance evaluation indicator Q is established to discuss the estimation results of the two methods, and the better RUL estimation result is selected for the diesel generator gearbox.

In addition, for a mechanical system, we will use the both methods and then prefer a more suitable result. Performance evaluation indicator Q is used to measure which result is better, they are some index like deviation of estimation in Appendix B. The two methods make the fusion process from different perspectives and take into account influencing factors comprehensively, so there is no need to fuse the two method’ results.

## 3. Experimental Results and Comparative Analysis

**Figure 8:**
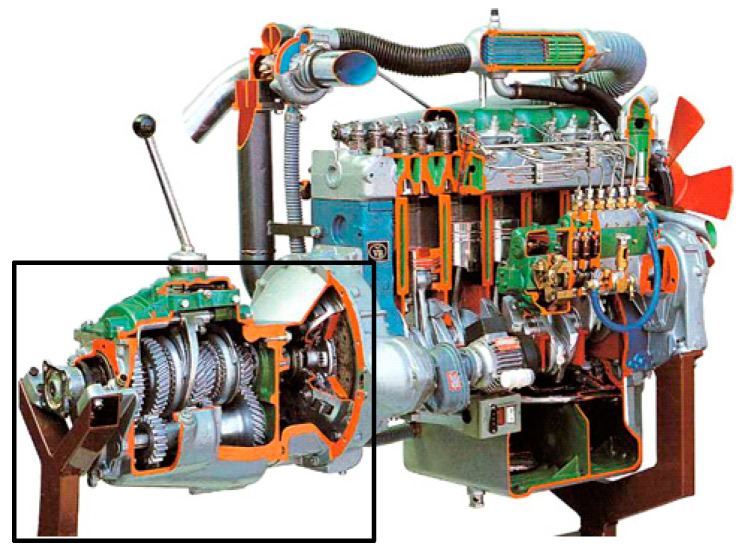
Typical diesel engine (Inside the black frame is a gearbox).

In this paper, the *RUL* of a diesel generator gearbox is studied by analyzing the vibration signals of a gearbox shell surface as Figure 8 showed. Data comes from the High Stress Accelerated Life Test of a certain type of heavy high-speed vessel diesel manufactured by the China Shipbuilding Industry Corporation (CSIC), which is collected from the gearbox Monoblock’s accelerometers. The number of teeth of the drive pinion is 17, and the number of teeth of the driven bull gear is *75*. The input shaft bearing has a pitch diameter of 60 mm, a rolling element diameter of 19.05 mm, and six steel balls; the output shaft bearing has a diameter of 95 mm, a rolling element diameter of 22.25 mm, and eight steel balls. The data were recorded every 5 or 10 min at a sampling rate of 20 KHz. Four sets of diesel generator gearbox data were recorded during the life-cycle degradation process in Table 1. *GU1, GU2, GU3,* and *GU4* all belong to the same type of component of the system, which are of similar working environments and operating conditions.

**Figure 9:**
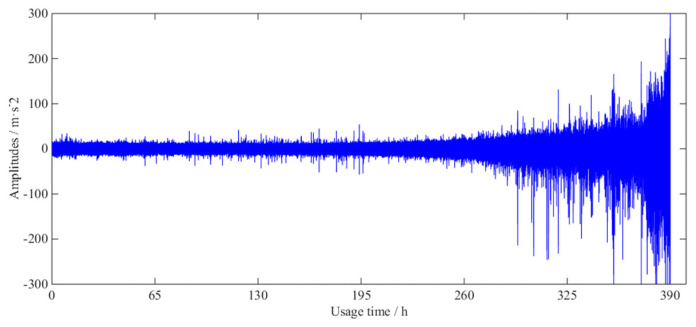
Vibration signal diagram in the life-cycle degradation process.

Figure 9 depicts the whole vibration signal in a gearbox lifecycle. The amplitude of the vibration signal increases gradually until the gear box fails to work properly.

### 3.1. Parameters System: Gearbox Performance Degradation Data

**Figure 10:**
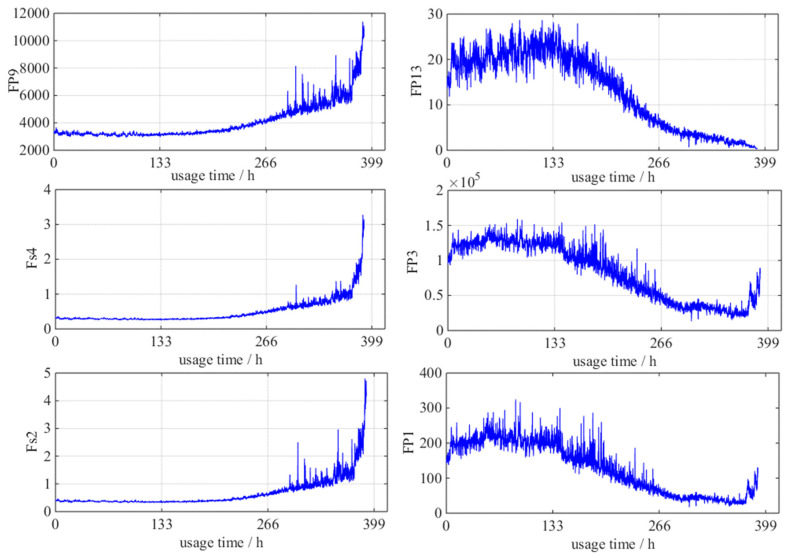
Life-cycle diagrams of six selected performance damage indicators.

With the theory in Section 2.1 and Section 2.2, the evaluation result of 25 damage indicators is showed in Table 2. According to the calculation result of Equation (7), the weights are $\omega_{1}$ = 0.2, $\omega_{2}$ = 0.5, and $\omega_{3}$ = 0.3, respectively. According to the Section 2.2, the first six damage indicators (*F_p_*_9_, *F_p_*_13_, *F_s_*_4_, *F_p_*_3_, *F_s_*_2_, and *F_p_*_1_) ranked from large to small according to the *W* value are selected to construct the performance damage indicator system of the diesel generator gearbox. They will be the input of two RUL estimation methods. Figure 10 shows the life-cycle trajectories of *F_p_*_9_, *F_p_*_13_, *F_s_*_4_, *F_p_*_3_, *F_s_*_2_, and *F_p_*_1_.

**Figure 11:**
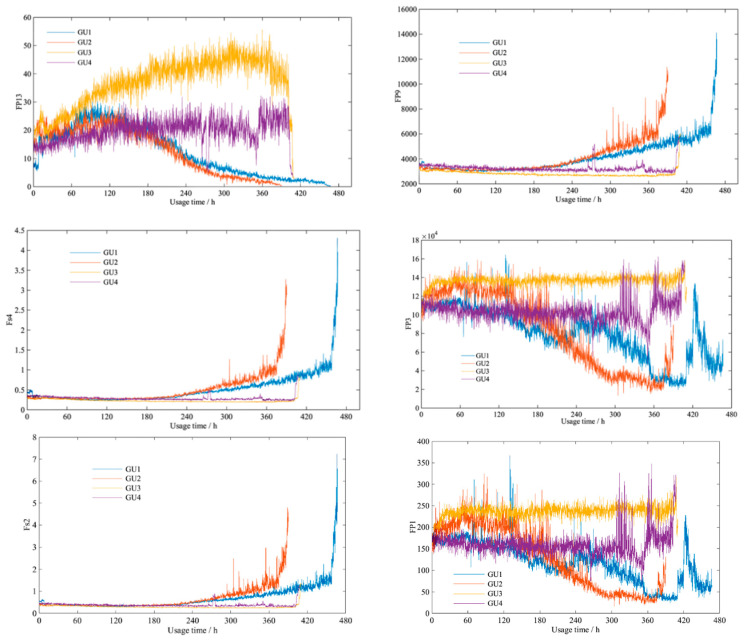
Curves of the six performance damage indicators from different samples.

After establishing the gearbox performance damage indicator system [*F_p_*_9_, *F_p_*_13_, *F_s_*_4_, *F_p_*_3_, *F_s_*_2_, *F_p_*_1_], the performance damage indicator data set of four samples (GU1 to GU4) is calculated. Figure 11 indicates that the curves of the same performance damage indicator from different samples have similar states. This proves that the gearbox registers a similar degradation trajectory in line with the running state and environment, which provides strong practical evidence for the subsequent *RUL* prediction based on multi-parameter and multi-sample similarity fusion. On the other hand, the different characters of *F_P_*_13_*, F_P_*_3_*,* and *F_P_*_1_ exactly reflect the different performance degradation trajectories of four samples. By selecting samples with different performance degradation, the verification of experience could be more convincing. In addition, in the aspect of the sample, *F_P_*_13_*, F_P_*_3_*,* and *F_P_*_1_ from a same sample have similar degradation trajectories, and the amplitude ranges are also so similar. This proves that these three parameters could actually reflect the performance degradation and should be selected for *RUL* prediction.

Considering the running time and data features, this study sets Sample *GU1* as the test sample and *GU2, GU3,* and *GU4* as reference samples to prove the validity of multi-parameter and multi-sample similarity fusion.

### 3.2. RUL Prediction Results

(1) Results based on multi-parameter similarity fusion

**Figure 12:**
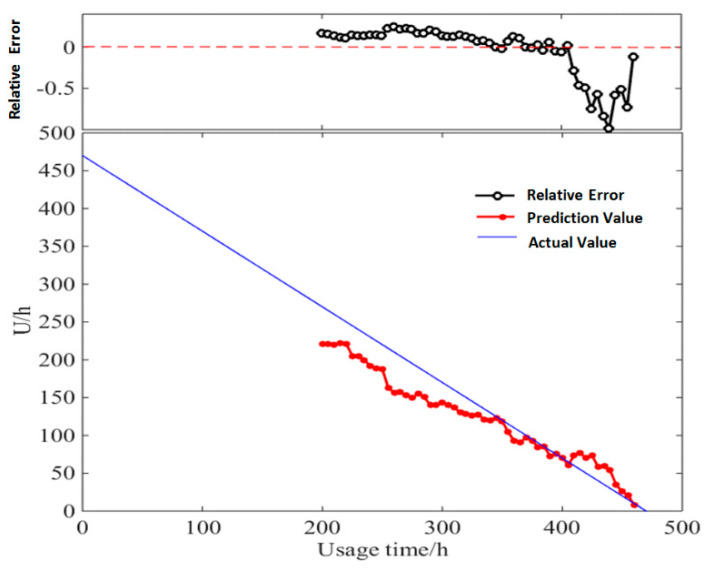
Actual values and predicted values of *RUL* based on multi-parameter similarity fusion with Euclidean distance.

**Figure 13:**
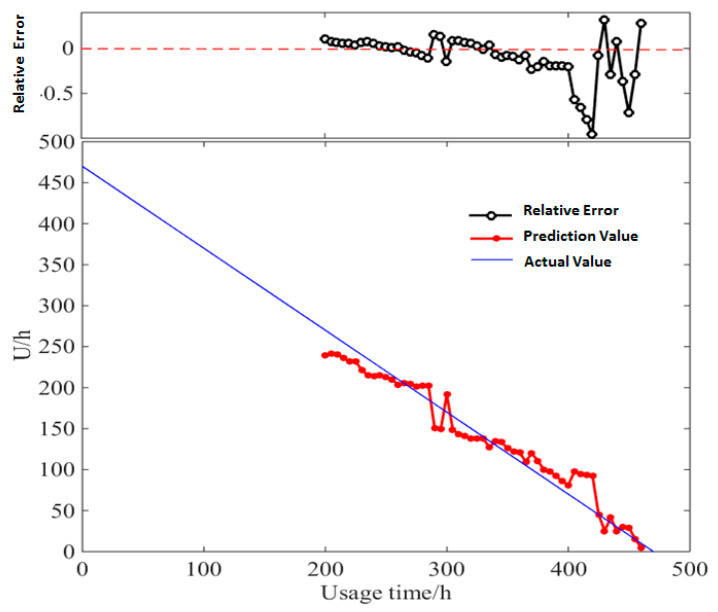
Actual values and predicted values of *RUL* based on multi-parameter similarity fusion with Dynamic Time Warping Distance (*DTWD)*.

This study unrolled the prediction of a diesel generator’s data starting from the point of 200 h, with the time window *D* of 30. The details of *RUL* prediction result based on multi-parameter similarity fusion with Euclidean distance/*DTWD* are shown in Table A3 and Table A4 of Appendix B. Figure 12 and Figure 13 show the relative error between the actual values and predicted values of *RUL*.

In *RUL* prediction based on multi-parameter similarity fusion with *DTWD*, the relative error between the predicted values and the actual values ranges from −0.88% to −95.82%. Except for very few points with large errors, the relative errors of most of the predicted values are below 30%, which could obtain more accurate values than traditional Euclidean distance.

(2) Result based on multi-sample similarity fusion

**Figure 14:**
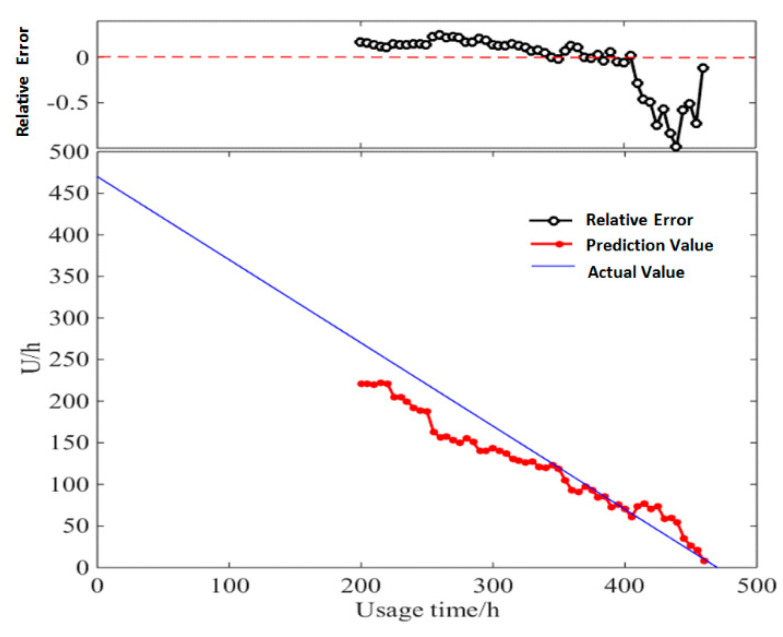
Actual values and predicted values of *RUL* based on multi-sample similarity fusion with Euclidean distance.

**Figure 15:**
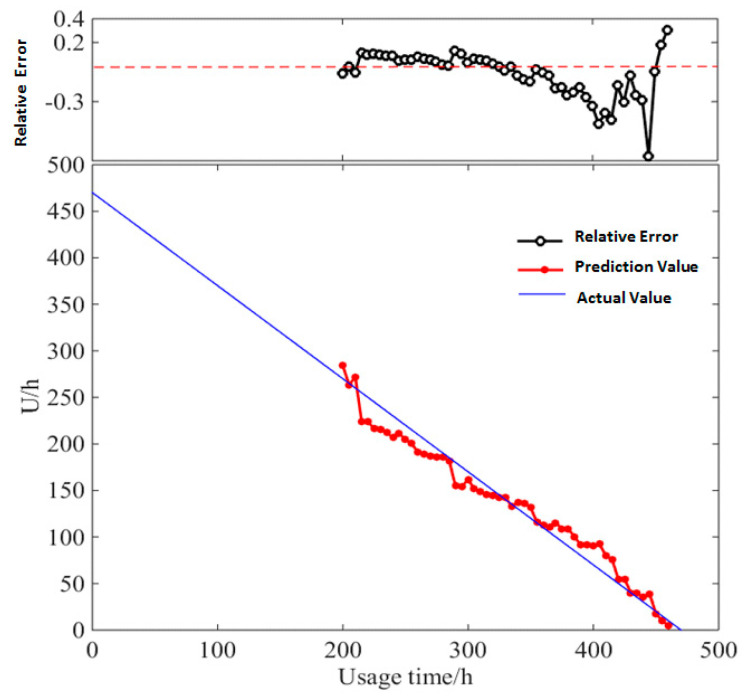
Actual values and predicted values of *RUL* based on multi-sample similarity fusion with *DTWD*.

The *RUL* estimation values based on multi-sample similarity fusion during the life-cycle degradation process are shown in Table A5 and Table A6 of Appendix B. Figure 14 and Figure 15 show the relative error between the actual values of *RUL* and the predicted values with Euclidean distance /*DTWD*.

With *RUL* prediction based on multi-sample similarity fusion, the relative error between the predicted values and the actual values ranges from −0.35% to −76%. Except for very few points with large errors, the overall relative error is controlled below 30%, which has a better prediction accuracy than the *RUL* prediction result based on multi-parameter similarity fusion.

### 3.3. Comparative Analysis with Single-Parameter RUL Prediction

Unlike methods of single-parameter similarity fusion, the method of multi-parameter similarity fusion generates a combination of results predicted by multiple parameters. In order to prove the validity and rationality of the model, a performance degradation curve is established upon each and every one of the reference samples’ parameters. The calculation adopts that of the single-parameter similarity prediction method and the weight value calculation process of different reference samples is the same as above. The test data set contains six performance damage indicators of Sample *GU1*: *F_p_*_9_, *F_p_*_13_, *F_s_*_4_, *F_p_*_3_, *F_s_*_2_, and *F_p_*_1_. They are compared to parameters *F_p_*_9_, *F_p_*_13_, *F_s_*_4_, *F_p_*_3_, *F_s_*_2_, and *F_p_*_1_ of Sample *GU2, GU3*, and *GU4* to determine the *RUL*.

In this study, Principal Component Analysis (*PCA*) technology is used to integrate elements of the performance degradation index system [30]. The first principal component *PCA*-1 and the second principal component *PCA*-2 were extracted respectively to conduct *RUL* prediction through the single-parameter life *RUL* prediction method [31]. This paper takes the life cycle data set of Sample GU1 as an example. Through *PCA* of its six performance damage indicators, we get the *KMO* of 0.748, higher than 0.5, indicating that the six parameters are suitable for dimensionality reduction processing.

**Figure 16:**
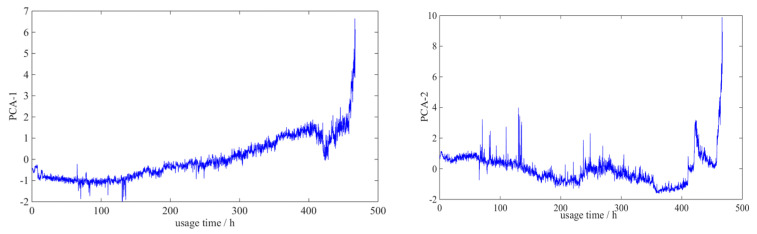
First- and second-order principal component of Sample GU’s performance damage indicator system.

The curve of the first-order principal component and second-order principal component of the performance damage indicator system of Sample GU1’s life cycle data is shown in Figure 16.

The single-parameter *RUL* prediction results of the first-order performance principal component *PCA*-1 and the second-order principal component *PCA*-2 are shown in Table 3.

It can be seen from the table that there are significant differences in the prediction effects of the six parameters in the diesel generator gearbox performance degradation index system. The prediction accuracy of *F_p_*_13_ and *F_s_*_4_ is higher than the rest, whereas the prediction of all the six first-order principal components of *PCA*-1 is more accurate than that of a single-parameter. The single-parameter similarity *RUL* prediction registers a poorer performance. In summary, the multi-parameter fusion-based *RUL* prediction method proposed in this study has certain advantages and effectiveness.

### 3.4. Comparative Analysis with AI-Based RUL Method: MSVM

Research on *RUL* prediction based on artificial intelligence has also been developed, such as Bayesian methods, which are deep learning methods. This paper uses the multivariable support vector machine (*MSVM*) for comparative analysis. *MSVM* fully considers the interaction and constraints between multiple variables, and realizes the maximum mining of potential information for small sample data. According to Section 3.1, *F_p_*_9_*, F_p_*_13_*, F_s_*_4_*, F_p_*_3_*, F_s_*_2_*,* and *F_p_*_1_ are selected as the input of *MSVM*, and a regression function is constructed:

(20) $$f(x)=(w\cdot x)+b,(w\in R^{n},b\in R)$$

*w* and *b* can be obtained by solving the optimum solution of the following equation:

(21) $$\begin{array}{l} \min\frac{1}{2}\|w{\|}^{2}+C\sum_{i=1}^{n}\left(\xi_{i}+\xi_{i}^{*}\right)\\ \mathrm{with}:\langle w\cdot x_{i}\rangle +b-y_{i}\leq \xi_{i}+\varepsilon ;y_{i}-\langle w\cdot x_{i}\rangle -b\leq \xi_{i}^{*}+\varepsilon \;i=1,2,\cdots ,n;\zeta_{i},\zeta_{i}^{*}\geq 0 \end{array}$$

*C* is a penalty factor, $\zeta_{i},\zeta_{i}^{*}$ are relaxation factors, and ε is an unsensitive factor. When the data set shows a nonlinear relationship, a kernel function is introduced into the *SVM* operation to map the original data into the high-dimensional feature space. The Radial Basis Function (*RBF*) and Poly kernel function are as follows:

(22) $$K\left(x_{i},x_{j}\right)=\exp\left(\frac{-{\left\|x_{i}-x_{j}\right\|}^{2}}{2p^{2}}\right)$$

P is the index of *RBF*. The Lagrangian function is introduced to transform the optimization problem into a convex quadratic programming problem. $\alpha_{i}$, $\alpha_{i}^{*}$ are Lagrangian multipliers.

(23) $$\begin{array}{l} \max W\left(\alpha_{i},\alpha_{i}^{*}\right)=-\frac{1}{2}\sum_{i,j=1}^{n}\left(\alpha_{i}-\alpha_{i}^{*}\right)\left(\alpha_{j}-\alpha_{j}^{*}\right)K\left(x_{i},x_{j}\right)-\varepsilon \sum_{i=1}^{n}\left(\alpha_{i}-\alpha_{i}^{*}\right)+\sum_{i=1}^{n}y_{i}\left(\alpha_{i}-\alpha_{i}^{*}\right)\\ with:\sum_{i=1}^{n}\left(\alpha_{i}-\alpha_{i}^{*}\right)=0;0\leq \alpha_{i}\leq C;0\leq \alpha_{i}^{*}\leq C(i=1,2,\cdots ,n) \end{array}$$

The calculation results of the comparative analysis are shown in Table 4.

Table 4 indicates that the prediction accuracy of multi-sample similarity fusion is higher than multi-parameter similarity fusion concerning the prediction’s average relative error, and the two methods’ *MAPE* are both lower than *MSVM*, validating the effectiveness of the proposed method compared with the *AI*-based method. In addition, the proposed *DTWD*-based algorithm performs better than the traditional Euclidean distance.

In parameter similarity fusion, *RUL* values predicted by the same performance damage indicators are integrated to calculate the *RUL* of the test sample; while in sample similarity fusion, the *RUL* values of samples are integrated on the basis of performance damage indicators carried by each sample.

**Figure 17:**
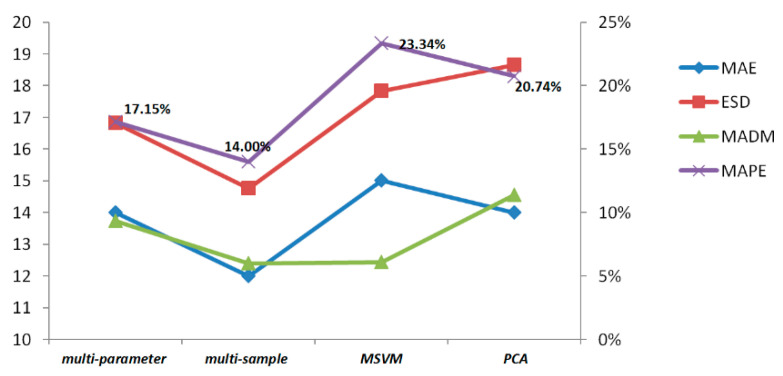
Comparison of four *RUL* prediction methods.

Multi-parameter and multi-sample methods are similar in calculation, but differ in some respects. Multi-parameter similarity fusion depends more on parameters’ feedback on the performance degradation process, while multi-sample similarity fusion relies on the sample data that is similar to the life-cycle trajectory in the gearbox running process. The more similar the test samples are with reference samples in terms of operating methods, conditions, and load environments, the larger the weight value that can be obtained, and the closer the *RUL* prediction value is to the actual value. Experimental results of the comparison are shown in Figure 17.

### 3.5. Limitations and Future Work

This paper proposes an *RUL* prediction model based on multi-parameter and multi-sample fusion, and has verified its effectiveness through analyzing a certain type of heavy high-speed diesel generator manufactured by an affiliate of *CSIC*. The results show that the proposed method is superior to previous studies in terms of the prediction accuracy. However, there are still some limitations in several respects. First, this paper verifies the proposed model with the diesel generator gearbox as an example, but further efforts should be devoted to testing broader gearbox equipment and even the mechanical rotating equipment. Second, this study does not classify types of malfunction at the termination and identify the degradation trend at different stages. Future researches can focus more on *RUL* under different malfunctions, grouping and decomposing the performance degradation process to identify test samples’ running stages, and refining the *RUL* prediction problems and models. Third, the research is conducted on the vibration signal of the diesel generator gearbox. To develop a more comprehensive *RUL* prediction method, future research should incorporate more data sources, such as performance parameters and environmental parameters.

## 4. Conclusions

This paper takes a certain type of heavy high-speed diesel generator as the study case. In the first step, through extracting time and frequency domain features of the original vibration and fuzzy filtering based on approximate entropy variance, the diesel generator performance damage indicators system is established. Next, this paper analyses the four core elements of similarity-based *RUL* prediction and establishes *DTWD* as the similarity measurement function. Then, we propose the methods of multi-parameter similarity fusion and multi-sample similarity fusion. Based on the two methods, the performance comparison research is carried out. The experimental results show that the *MAPE* values of the two *RUL* prediction methods proposed here are below 14%, which are lower than *MSVM*’s and *PCA*’s. This fully validates the effectiveness of the proposed method for predicting the *RUL*.And the *RUL* prediction based on the dynamic time bending distance function in the sample similarity fusion has the best accuracy which is below 10%. The similarity-based *RUL* prediction method has the merit of avoiding establishing a system degradation model, and is simple and practical. Moreover, it fully employs effective information provided by vibration signals, considers multiple parameters that can reflect performance degradation, and conducts a comparative analysis of multiple samples. The predicted results are stable as experimental results showed.

In summary, the innovations of this article are mainly as follows:

(1) We put forward the idea of similarity fusion with multi-parameter and multi-sample methods, and established the *RUL* prediction model. The performance degradation process is multi-dimensional and multifaceted. Multi-parameter similarity fusion takes full consideration of multiple parameters of vibration signals and a whole performance degradation process. Hence, a more comprehensive and accurate prediction is achieved. In contrast, multi-sample similarity fusion considers multiple samples with life-cycle degradation. By integrating *RUL* prediction values calculated by damage indicators carried with those samples, we improve the stability and credibility of *RUL* prediction; the *MAPE* is reduced to less than 14%, the *MSE* less than 220, the *MADM* less than 13.
(2) The *DTWD*-based nonlinear dynamic programming algorithm is established as the distance measure of similarity in *RUL* prediction. In the time series analysis, it performed better than the traditional Euclidean distance, the average relative errors of DTWD is 17% less than Euclidean distance.
(3) After time domain and frequency domain features extraction, we proposed approximate entropy variance (Aev) for low-pass filtering to remove signal noise.

## Figures and Tables

**Table 1:** Vibration data sets for the diesel generator gearbox.

Gearbox No.	Service Time (Unit: h)	Record Interval (Unit: min)
GU1	467	5 or 10
GU2	390	5 or 10
GU3	410	5 or 10
GU4	408	5 or 10

**Table 2:** Evaluation results of time-domain and frequency-domain damage indicators.

Damage Indicators	Corr	Mon	Rob	W	Ranking	Damage Indicators	Corr	Mon	Rob	W	Ranking
*F_s_*_1_	0.0723	0.0060	0.3936	0.13554	25	*F_p_*_4_	0.6379	0.0496	0.9491	0.43711	10
*F_s_*_2_	0.7374	0.0641	0.9418	0.46207	5	*F_p_*_5_	0.5585	0.0641	0.8725	0.4055	14
*F_s_*_3_	0.5224	0.0675	0.9084	0.41075	13	*F_p_*_6_	0.9134	0.0051	0.9016	0.45571	8
*F_s_*_4_	0.7452	0.0675	0.9514	0.46821	3	*F_p_*_7_	0.1305	0.0436	0.5069	0.19997	24
*F_s_*_5_	0.7440	0.0051	0.4542	0.28761	22	*F_p_*_8_	0.1091	0.0017	0.9956	0.32135	20
*F_s_*_6_	0.5463	0.0009	0.9797	0.40362	15	*F_p_*_9_	0.8201	0.0513	0.9696	0.48055	1
*F_s_*_7_	0.5228	0.0009	0.8678	0.36535	18	*F_p_*_10_	0.4615	0.0188	0.9928	0.39954	16
*F_s_*_8_	0.6418	0.0239	0.8301	0.38934	17	*F_p_*_11_	0.5650	0.0239	0.9917	0.42246	11
*F_s_*_9_	0.7128	0.0474	0.8501	0.42129	12	*F_p_*_12_	0.0447	0.0265	0.9950	0.32069	21
*F_s_*_10_	0.8328	0.0248	0.8696	0.43984	9	*F_p_*_13_	0.8896	0.0615	0.8985	0.47822	2
*F_p_*_1_	0.9135	0.0085	0.9071	0.45908	6	*F_p_*_14_	0.4418	0.0094	0.5156	0.24774	23
*F_p_*_2_	0.9135	0.0085	0.9071	0.45908	7	*F_p_*_15_	0.3534	0.0581	0.8413	0.35212	19
*F_p_*_3_	0.9105	0.0077	0.9222	0.46261	4						

**Table 3:** Comparison with single-parameter Remaining Useful Life (*RUL)* prediction.

Parameter	Performance Evaluation Indicators Q				
	MAE	MSE	MAPE	ESD	MADM
F*_p_*_9_	23	751.86	36.51%	24.78	21.10
F*_p_*_13_	20	709.71	20.30%	26.45	19.97
F*_s_*_4_	21	676.81	21.35%	21.98	18.92
F*_p_*_3_	41	4142.53	79.74%	63.75	40.72
F*_s_*_2_	23	1014.17	40.64%	25.22	19.34
F*_p_*_1_	38	3023.67	69.93%	54.82	38.20
*PCA*-1	13	241.22	18.79%	15.39	13.05
*PCA*-2	16	490.60	22.69%	21.90	16.02

**Table 4:** Results of different prediction methods.

*RUL* Prediction Method	Similariy Measure	Performance Evaluation Indicators *Q*				
		MAE	MSE	MAPE	ESD	MADM
multi-parameter similarity fusion	Euclidean distance	23	808.81	30.90%	27.78	22.56
	*DTWD* function	14	292.11	17.15%	16.82	13.73
multi-sample similarity fusion	Euclidean distance	22	684.30	21.43%	22.41	19.35
	*DTWD* function	12	219.37	14.00%	14.76	12.39
*MSVM*	Euclidean distance	19	351.02	23.34%	17.83	12.24
	*DTWD* function	17	287.34	20.14%	16.21	11.45

## Appendix A

In Table A1, *F_ss_* represents the time domain parameter of ***s***, and ***x_i_*** represents the amplitude of the vibration signal collected by the gearbox sensor within a certain period (*i* = 1, 2, …, *N*_0_), where *N*_0_ is the quantity of data points collected within the period.

**Table A1:** Time domain damage indicators of the gearbox vibration signal.

Damage Indicators	Feature Symbol	Equation	Implication
Average value	$F_{s1}$	$F_{s1}=\frac{1}{N_{0}}\sum_{i=1}^{N_{0}}x_{i}$	Average energy value of gearbox vibration within a certain period
Mean square	$F_{s2}$	$F_{s2}=\sqrt{\frac{1}{N_{0}}\sum_{i=1}^{N_{0}}x_{i}{}^{2}}$	Better manifesting performance degradation trend of gear and bearing [32,33,34]
Mean-square amplitude	$F_{s3}$	$F_{s3}={\left[\frac{1}{N_{0}}\sum_{i=1}^{N_{0}}\sqrt{\left|x_{i}\right|}\right]}^{2}$	Sensitive to larger amplitude change [35]
Absolute average	$F_{s4}$	$F_{s4}=\frac{1}{N_{0}}\sum_{i=1}^{N_{0}}\left|x_{i}\right|$	Calculating absolute value before calculating averages, which can avoid positive-negative offset
Skewness index	$F_{s5}$	$F_{s5}=\frac{1}{N_{0}}\sum_{i=1}^{N_{0}}x_{i}{}^{3}$	Measuring asymmetry of vibration signals
Waveform index	$F_{s6}$	$F_{s6}=\frac{\sqrt{\frac{1}{N_{0}}\sum_{i=1}^{N_{0}}x_{i}^{2}}}{F_{s4}}$	Representing deviated and inclined value between present vibration signal and sine wave
Pulsatility index	$F_{s7}$	$F_{s7}=\frac{\max(x_{i})}{\frac{1}{N}\sum_{i=1}^{N_{0}}\left|x_{i}\right|}$	Manifesting stability and destruction level of gearbox’s degradation and malfunction [36]
Kurtosis index	$F_{s8}$	$F_{s8}=\frac{\frac{1}{N_{0}}\sum_{i=1}^{N_{0}}x_{i}^{4}}{{\left(\sqrt{\frac{1}{N_{0}}\sum_{i=1}^{N_{0}}x_{i}^{2}}\right)}^{4}}$	Measuring “bending and arching” level of vibration signal
Peak-peak value	$F_{s9}$	$F_{s}{}_{9}=max\left(x_{i}\right)-min\left(x_{i}\right)$	Reflecting impact vibration resulted from malfunction
Margin index	$F_{s10}$	$F_{s10}=\frac{\max(x_{i})}{F_{s3}}$	Reflecting Abrasion level of gear and bearing [35]

In Table A2, The spectrum of the original signal *x_i_* collected within a certain period is represented as s*_j_*, where *j* = 1, 2, …, J. *J* is the spectral line quantity of the spectrum, f_j_ represents the frequency value of the j-th line, and F_pk_ below represents the value of the k-th frequency domain damage indicator F_p_.

**Table A2:** Frequency domain features of the vibration signal.

Feature Symbol	Equation	Feature Symbol	Equation	Feature Symbol	Equation
$F_{p1}$	$\frac{\sum_{j=1}^{J}{\bar{s}}_{j}\cdot s_{j}}{2\pi \sum_{j=1}^{J}s_{i}{}^{2}}$	$F_{p2}$	$\frac{\sum_{j=1}^{J}{\bar{s}}_{j}{}^{2}}{4\pi^{2}\sum_{j=1}^{J}s_{i}{}^{2}}$	$F_{p3}$	$F_{p}{}_{2}-F_{p}{{}_{1}}^{2}$
$F_{p4}$	$\frac{\sum_{j=1}^{J}s_{j}}{J}$	$F_{p5}$	$\frac{\sum_{j=1}^{J}{\left(s_{j}-F_{p4}\right)}^{2}}{J-1}$	$F_{p6}$	$\frac{\sum_{j=1}^{J}{\left(s_{j}-F_{p4}\right)}^{3}}{J{\left(\sqrt{F_{p5}}\right)}^{3}}$
$F_{p7}$	$\frac{\sum_{j=1}^{J}{\left(s_{j}-F_{p4}\right)}^{4}}{JF_{p5}{}^{2}}$	$F_{p8}$	$\frac{\sum_{j=1}^{J}f_{j}s_{j}}{\sum_{j=1}^{J}s_{j}}$	$F_{p9}$	$\sqrt{\frac{\sum_{j=1}^{J}{\left(f_{j}-F_{p8}\right)}^{2}s_{j}}{J}}$
$F_{p10}$	$\sqrt{\frac{\sum_{j=1}^{J}f_{j}^{2}s_{j}}{\sum_{j-1}^{J}s_{j}}}$	$F_{p11}$	$\sqrt{\frac{\sum_{j=1}^{J}f_{j}^{4}s_{j}}{\sum_{j-1}^{J}f_{j}^{2}s_{j}}}$	$F_{p12}$	$\frac{\sum_{j=1}^{J}f_{j}^{2}s_{j}}{\sqrt{\sum_{j=1}^{J}s_{j}\sum_{j=1}^{J}f_{j}^{4}s_{j}}}$
$F_{p13}$	$\frac{F_{p9}}{F_{p8}}$	$F_{p14}$	$\frac{\sum_{j=1}^{J}{\left(f_{j}-F_{p8}\right)}^{3}s_{j}}{JF_{p9}{}^{3}}$	$F_{p15}$	$\frac{\sum_{j=1}^{J}{\left(f_{j}-F_{p8}\right)}^{4}s_{j}}{JF_{p9}{}^{4}}$

## Appendix B

(1) Calculation of *Corr*, *Mon* and *Rob*

(A1) $$Corr\left(X,T\right)=\frac{\left|K\sum_{k}X_{T}(t_{k})t_{k}-\sum_{k}X_{T}(t_{k})\sum_{k}t_{k}\right|}{\sqrt{\left[K\sum_{k}X_{T}{\left(t_{k}\right)}^{2}-(\sum_{k}X_{T}(t_{k}){)}^{2}\right]\left[K\sum_{k}t_{k}{}^{2}-(\sum_{k}t_{k}{)}^{2}\right]}}$$

(A2) $$Mon(x)=\frac{1}{K-1}\left|\sum_{k}\delta (X_{T}(t_{k+1})-X_{T}(t_{k}))-\sum_{k}\delta (X_{T}(t_{k})-X_{T}(t_{k+1}))\right|$$

(A3) $$Rob(X)=\frac{1}{K}\sum_{k}\exp(-\left|\frac{X_{R}(t_{k})}{X(t_{k})}\right|)$$

In those equations, *K* represents the total number of time series and δ( ) represents the unit step function. When the value of the independent variable in parentheses is larger than 0, the value of δ( ) is 1; otherwise, the value of δ( ) is 0.They are all distributed in the range of [0, 1] and positively correlated with time domain features and frequency domain features.

(2) The performance evaluation indicator Q

**Mean Absolute Error (MAE)**: *B* is the start time of the test sample’s prediction, *E* is the end time of prediction, and *i* is the time point of prediction. *Δ(i)* represents the difference between the predicted value and actual value of the *i*-th prediction. The smaller the MAE is, the higher the prediction accuracy is.

(A4) $$MAE=\frac{\sum_{i=B}^{E}\left|\Delta \left(i\right)\right|}{E-B+1}$$

**Mean Squared Error (MSE):**

(A5) $$MSE=\frac{1}{E-B+1}\sum_{i=1}^{E-B+1}\Delta {\left(i\right)}^{2}$$

**Mean Absolute Percentage Error (MAPE)****:** The concept of Relative Error is introduced in this paper considering the difference between the predicted value and actual value.

(A6) $$MAPE=\frac{1}{E-B+1}\sum_{i=1}^{E-B+1}\left|\frac{100\Delta (i)}{U\left(i\right)}\right|$$

**Error Standard Deviation (ESD)****:** This reflects fluctuation of the error value. The smaller the value is, the more stable the gearbox is.

(A7) $$M=\frac{1}{E-B+1}\sum_{i=1}^{E-B+1}\Delta \left(i\right)$$

(A8) $$ESD=\sqrt{\frac{\sum_{i=1}^{E-B+1}{\left(\Delta \left(i\right)-M\right)}^{2}}{E-B}}$$

**Error Standard Deviation (ESD)****:***M**e* denotes the median of the error value. MADM reflects the deviation degree of the error value from the median value, which applies to cases where the error value does not conform to Normal Distribution.

(A9) $$MADM=\frac{1}{E-B+1}\sum_{i-1}^{E-B+1}\left|\Delta \left(i\right)-Me\right|$$

(3) The pseudocode of *DTWD* (Algorithm A1)

**Algorithm A1: Main: calculate *DTWD***
**Input**: *time series A (array* [1, *l*]*), B(array* [1,*k*]*)*
**Output**: *DTWD*
**(1)** Matrix D = A^T^ B.
**(2) Set** constraint condition:
Boundedness, Boundary conditions, Continuity, and Monotonicity
**(3)** D_twd_ = 0
**(4) FOR***i:* = 1:l **DO**
*DTWD* [*i*, 0]: = ∞
**(5) FOR***i:* = 1:l **DO**
*DTWD* [0, *i*]: = ∞
**(6)** DTW [0, 0]: = 0
**(7) FOR***i* = 1: l **DO**
**{ FOR***j* = 1: k **DO**
$d\left(i,j\right)={\left\|i-j\right\|}_{p}$
cost:=d(A[i],B[j])
*DTWD* [*i*,*j*]: = cost + **minimum** (*DTWD* [*i* − 1,j], *DTWD* [*i*,*j* − 1], *DTWD* [*i* − 1,*j* − 1])
}
**(8) return** *DTWD*

The details of *RUL* prediction result based on multi-parameter similarity fusion with Euclidean distance/*DTWD*.

**Table A3:** *RUL* prediction results based on multi-parameter similarity fusion with Euclidean distance.

NO.	*RUL*		Error	Relative Error (%)	NO.	*RUL*		Error	Relative Error (%)
	Actual Value	Predicted Value				Actual Value	Predicted Value		
1	267	348	−81	−30.35	28	132	128	4	2.97
2	262	238	24	9.17	29	127	137	−10	−7.50
3	257	351	−94	−36.54	30	122	135	−13	−10.33
4	252	235	17	6.91	31	117	128	−11	−9.35
5	247	236	12	4.67	32	112	115	−3	−2.90
6	242	227	15	6.39	33	107	122	−15	−13.69
7	237	223	14	6.05	34	102	129	−27	−26.31
8	232	218	14	6.03	35	97	116	−19	−19.45
9	227	208	19	8.36	36	92	111	−19	−20.36
10	222	209	13	5.83	37	87	113	−26	−30.06
11	217	207	10	4.73	38	82	103	−21	−25.65
12	212	203	9	4.12	39	77	94	−17	−21.66
13	207	166	42	20.08	40	72	96	−24	−33.46
14	202	167	36	17.61	41	67	92	−25	−37.67
15	197	163	34	17.20	42	62	97	−35	−55.75
16	192	161	31	16.34	43	57	90	−33	−57.49
17	187	161	26	14.14	44	52	97	−45	−86.60
18	182	156	26	14.06	45	47	95	−48	−101.49
19	177	154	23	12.77	46	42	71	−29	−68.44
20	172	152	20	11.77	47	37	71	−34	−91.10
21	167	149	18	10.65	48	32	65	−33	−102.14
22	162	150	12	7.35	49	27	63	−36	−131.98
23	157	147	10	6.16	50	22	51	−29	−130.16
24	152	142	10	6.36	51	17	39	−22	−127.27
25	147	143	4	2.66	52	12	15	−3	−23.33
26	142	143	−1	−0.31	53	7	15	−8	−109.38
27	137	143	−81	−4.34					

**Table A4:** *RUL* prediction results based on multi-parameter similarity fusion with *DTWD*.

NO.	*RUL*		Error	Relative Error (%)	NO.	*RUL*		Error	Relative Error (%)
	Actual Value	Predicted Value				Actual Value	Predicted Value		
1	267	239	28	10.36	28	132	127	5	3.95
2	262	242	20	7.67	29	127	135	−8	−6.38
3	257	240	17	6.52	30	122	134	−11	−9.38
4	252	237	15	6.13	31	117	126	−9	−7.55
5	247	232	15	6.13	32	112	122	−10	−8.69
6	242	232	10	4.28	33	107	121	−14	−12.84
7	237	222	15	6.52	34	102	110	−8	−7.56
8	232	215	17	7.22	35	97	120	−23	−23.53
9	227	214	13	5.73	36	92	111	−18	−19.95
10	222	215	7	3.05	37	87	100	−13	−14.76
11	217	213	4	1.85	38	82	98	−16	−19.31
12	212	210	2	1.00	39	77	92	−15	−19.27
13	207	204	4	1.72	40	72	86	−14	−19.21
14	202	206	−4	−1.81	41	67	81	−14	−20.64
15	197	205	−8	−3.89	42	62	98	−35	−56.96
16	192	201	−9	−4.66	43	57	95	−38	−66.05
17	187	202	−15	−8.12	44	52	94	−42	−79.67
18	182	202	−20	−11.01	45	47	92	−45	−95.82
19	177	150	27	15.23	46	42	45	−3	−7.55
20	172	149	23	13.36	47	37	25	12	31.81
21	167	191	−24	−14.59	48	32	42	−9	−29.06
22	162	148	14	8.44	49	27	25	2	7.94
23	157	143	14	9.00	50	22	30	−8	−36.89
24	152	141	11	7.10	51	17	30	−12	−71.91
25	147	138	9	5.94	52	12	16	−4	−28.81
26	142	138	5	3.19	53	7	5	2	27.88
27	137	138	−1	−0.88					

The details of *RUL* prediction result based on multi-sample similarity fusion with euclidean distance/*DTWD*.

**Table A5:** prediction results based on multi-sample similarity fusion with euclidean distance.

NO.	*RUL*		Error	Relative Error (%)	NO.	*RUL*		Error	Relative Error (%)
	Actual Value	Predicted Value				Actual Value	Predicted Value		
1	267	221	46	17.14	28	132	121	11	8.09
2	262	221	41	15.72	29	127	120	7	5.37
3	257	220	37	14.34	30	122	123	0	−0.38
4	252	222	30	11.89	31	117	119	−2	−1.60
5	247	221	26	10.43	32	112	105	8	6.75
6	242	205	37	15.25	33	107	93	14	13.36
7	237	204	33	13.78	34	102	91	11	10.94
8	232	199	33	14.07	35	97	97	0	−0.13
9	227	192	35	15.33	36	92	93	−1	−1.37
10	222	189	33	15.03	37	87	85	2	2.84
11	217	188	30	13.60	38	82	86	−3	−4.12
12	212	163	49	23.18	39	77	73	5	6.01
13	207	157	50	24.30	40	72	76	−4	−5.06
14	202	157	45	22.16	41	67	71	−4	−5.64
15	197	153	44	22.27	42	62	61	1	1.98
16	192	150	42	22.11	43	57	74	−17	−28.88
17	187	155	32	17.17	44	52	76	−24	−46.44
18	182	151	31	17.17	45	47	70	−23	−49.52
19	177	140	37	20.85	46	42	74	−31	−74.72
20	172	141	32	18.35	47	37	58	−21	−57.02
21	167	144	23	13.97	48	32	59	−27	−84.10
22	162	140	22	13.35	49	27	54	−27	−98.48
23	157	137	20	12.60	50	22	35	−13	−57.95
24	152	130	22	14.43	51	17	26	−9	−51.51
25	147	128	19	12.79	52	12	21	−9	−72.66
26	142	127	15	10.83	53	7	8	−1	−11.67
27	137	128	10	6.95					

**Table A6:** *RUL* prediction results based on multi-sample similarity fusion with *DTWD*.

NO.	*RUL*		Error	Relative Error (%)	NO.	*RUL*		Error	Relative Error (%)
	Actual Value	Predicted Value				Actual Value	Predicted Value		
1	267	284	−17	−6.40	28	132	133	0	−0.35
2	262	263	−1	−0.47	29	127	137	−10	−8.07
3	257	272	−15	−5.79	30	122	136	−14	−11.36
4	252	224	28	11.04	31	117	132	−15	−12.51
5	247	224	23	9.34	32	112	116	−4	−3.29
6	242	216	26	10.58	33	107	113	−6	−5.30
7	237	215	22	9.19	34	102	111	−9	−8.33
8	232	213	19	8.40	35	97	115	−18	−18.84
9	227	207	20	8.90	36	92	109	−17	−17.94
10	222	211	11	4.89	37	87	109	−21	−24.59
11	217	205	12	5.65	38	82	101	−18	−22.37
12	212	200	12	5.59	39	77	91	−14	−18.31
13	207	191	16	7.64	40	72	91	−19	−26.62
14	202	189	13	6.46	41	67	90	−23	−34.15
15	197	187	10	5.09	42	62	92	−30	−48.85
16	192	186	6	3.38	43	57	80	−23	−39.99
17	187	185	2	0.87	44	52	76	−24	−45.74
18	182	182	0	0.26	45	47	55	−8	−16.65
19	177	155	22	12.63	46	42	55	−13	−30.48
20	172	154	18	10.52	47	37	40	−3	−7.66
21	167	162	5	3.18	48	32	40	−8	−24.40
22	162	152	10	6.42	49	27	35	−8	−28.88
23	157	149	9	5.43	50	22	39	−17	−76.00
24	152	146	7	4.34	51	17	18	−1	−4.89
25	147	144	3	1.80	52	12	10	2	17.78
26	142	143	−1	−0.61	53	7	5	2	30.21
27	137	142	−5	−3.83					
